# Evaluation and Importance of Trauma

**DOI:** 10.5812/atr.7245

**Published:** 2012-08-21

**Authors:** Akbar Behdad

**Affiliations:** 1 Department of Surgery, Faculty of Medicine, Isfahan University of Medical Sciences, Isfahan, IR Iran

**Keywords:** Trauma, Research, Global Disease

## Editorial

Human life is considered of such an extensive value and importance in the human communities that directing all the facilities to fulfill this mission seems quite reasonable. All the health providers pay an immense attention to the holy Quran, as it could be understand from Maedeh surah “If anyone save a life, it would be as if he has saved the whole humanity” the human lives has a high value (1). It has been reported that over 5 million people die annually from injuries worldwide. Injuries as the third most important and frequent cause of overall mortality and the main cause of death among one to forty years old individuals, consist 12 percent of the global diseases (2). A large number of the world’s road fatalities occur in the Asia-Pacific region concerning to the increasing number of motor vehicles, it is predicted that the road trauma incidence is going to overtake stroke, tuberculosis, and prenatal conditions incidence by 2020 (3-5). As a result, the United Nations had designated 2011-2020 the Decade of Action on the Road Safety. Each severely injured patient should be given the best possible chance of independent recovery and treatment. This can only happen if highly effective strategies were taken from the earliest moment of injury to recovery. This in turn can only be achieved if pre-hospital; hospital and rehabilitation professionals consistently provide the best up-to-date caring services as well as facilities in align with an efficient systematic approach at the place where these services are provided. Although all people in different aging groups with various socioeconomic levels are at risk of trauma, however, the most efficient group in all societies are the most susceptible ones and this may even affect the integrity of a society as well as the future development. Considering the statement of the father of medicine, Hippocrates, indicating “Life is short, art long, opportunity fleeting, experiment treacherous, decision difficult” (6, 7), that is everybody’s duty to participate in management and trauma research activities to work harder in order to make a significant change, particularly in developing countries. In order to reach this achievement, research arrangements, stronger communication between the authorities, spreading information and guidelines and application of experiences of experts in different fields related to trauma may offer a milieu for better handling of the present ongoing disaster. Specialized peer reviewed journals in the field of trauma seems to be helpful in accomplishing the aforementioned mission through publication articles. This is a new approach in our country and we wish that it will be effective and lead to the highest levels of success.
